# Regulation of cellular proliferation, differentiation and cell death by activated Raf

**DOI:** 10.1186/1478-811X-7-8

**Published:** 2009-04-21

**Authors:** Gerald Thiel, Myriam Ekici, Oliver G Rössler

**Affiliations:** 1 Department of Medical Biochemistry and Molecular Biology, University of Saarland Medical Center, Homburg, Germany

## Abstract

The protein kinases Raf-1, A-Raf and B-Raf connect receptor stimulation with intracellular signaling pathways and function as a central intermediate in many signaling pathways. Gain-of-function experiments shed light on the pleiotropic biological activities of these enzymes. Expression experiments involving constitutively active Raf revealed the essential functions of Raf in controlling proliferation, differentiation and cell death in a cell-type specific manner.

## Introduction

**Figure 1:**
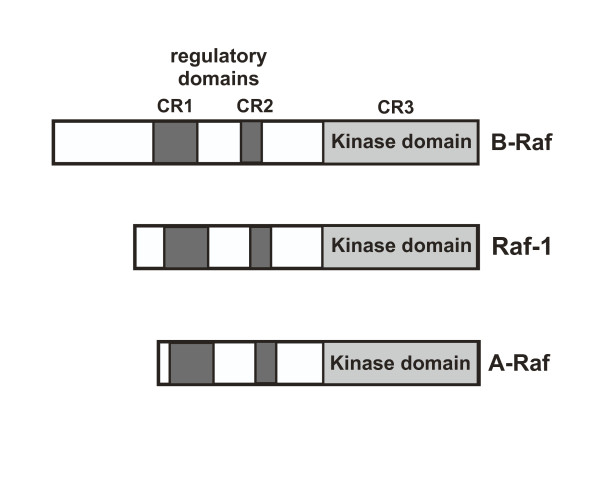
**Modular structure of Raf**. The Raf isoforms Raf-1, A-Raf and B-Raf share three conserved domains termed CR1, CR2 and CR3. CR1 contains a Ras-binding subdomain and a cysteine-rich subdomain, both required to bind to activated Ras (Ras-GTP) at the cell membrane. CR2 is rich in serine and threonine residues and negatively regulates the biological activity of the catalytic domain. This domain binds also regulatory 14-3-3 proteins. CR3 encompasses the protein kinase domain.

All three Raf isoenzymes are cytosolic serine/threonine protein kinases that exhibit a high degree of sequence similarity. The enzymes contain three domains termed CR1, CR2 and CR3. The N-terminal CR1 contains a Ras-binding subdomain and a cysteine-rich subdomain, both required to bind to activated Ras (Ras-GTP) at the cell membrane. CR2 is rich in serine and threonine residues and negatively regulates the biological activity of the catalytic domain. CR3 contains the catalytic protein kinase domain (figure 1).

Raf connects cellular stimulation with intracellular signaling pathways. Raf translocates to the plasma membrane as a result of receptor tyrosine kinase stimulation that leads to a subsequent activation of Ras. Following activation, Raf phosphorylates and activates mitogen-activated protein kinase (MAP) kinase (MEK) which in turn phosphorylates and activates the MAP kinases extracellular signal-regulated protein kinases ERK1 and ERK2 [1]. Raf functions therefore as a vital link between activated Ras and ERK. The activated protein kinases ERK1/2 are able to translocate into the nucleus and change the gene expression pattern via phosphorylation of gene regulatory proteins. Thus, activation of Raf is essential for activating the Raf/MEK/ERK signaling pathway and many functions attributed to Raf activation are executed by the subsequent activation of MEK and ERK. A microarray analysis confirmed that the transcriptional response to Raf activation almost completely depends on MEK activation [2]. In line with this, MEK is the only generally acknowledged substrate for Raf [1,3].

### Lessons from Raf-deficient mice

Gene ablation experiments involving the genes encoding the Raf isoforms Raf-1, A-Raf, and B-Raf revealed divergent phenotypes, indicating that Raf isoforms are not always able to compensate for each other. In particular, distinct essential functions are served by Raf-1 and B-Raf in embryonic development [4]. Nevertheless, a functional redundancy among the Raf family proteins exists and only phenotypes requiring the activity of a distinct Raf isoform are found. Inactivation of the Raf-1 and B-Raf-encoding genes revealed that Raf-1 and B-Raf play essential anti-apoptotic roles [5,6]. B-Raf is necessary for survival of embryonic motoneurons and sensory neurons [7]. Several review articles have been published that discuss these mouse models in detail [4,8-10]

### Gain-of-function mutants of Raf

Two strategies have been used to express constitutively active Raf. The translocation of Raf to the plasma membrane via binding to Ras-GTP is the key event in Raf activation [1]. Thus, a method to express a constitutively active Raf-1 relies in the tethering of Raf to the plasma membrane. This Raf-1 mutant termed Raf-CAAX carries at the C-terminus an isoprenylation sequence derived from K-Ras [11,12]. The artifical targeting of Raf-1 to the plasma membrane leads to an activation of the enzyme in a Ras-independent manner and shows 30-fold higher kinase activity in growth-factor-deprived cells.

**Figure 2:**
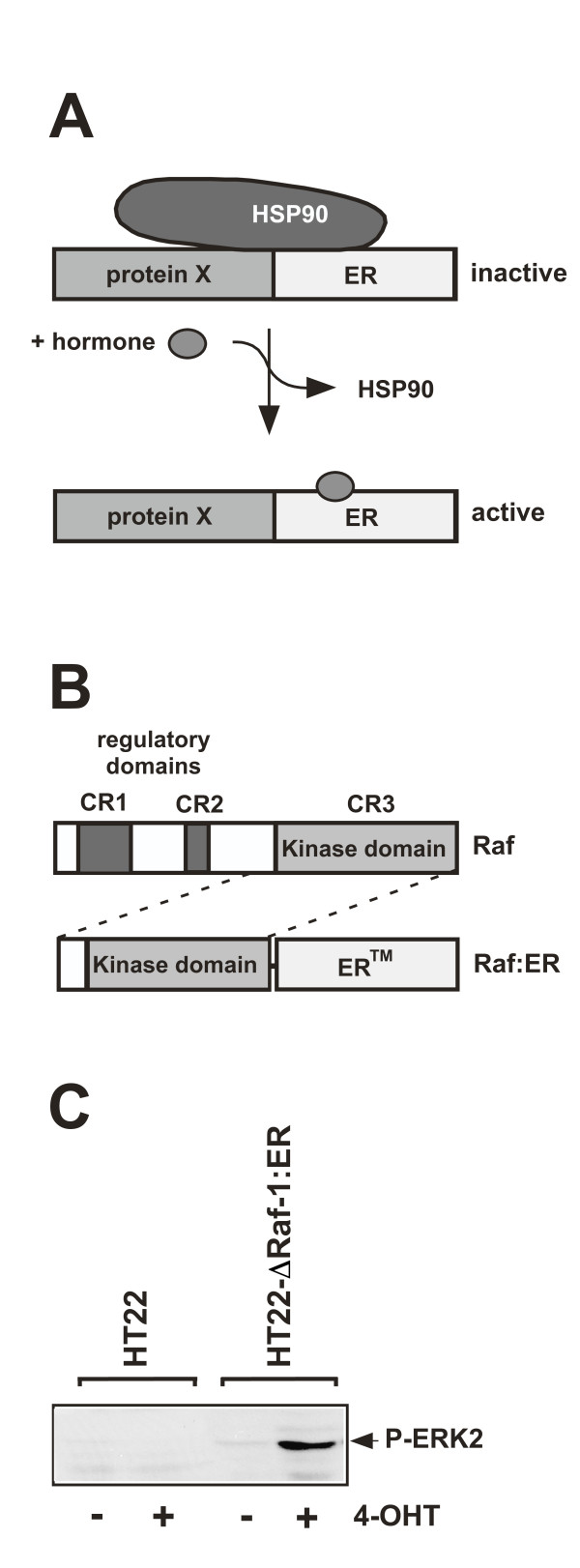
**Design and biological activity of a conditionally active forms of Raf**. (A) Strategy: A heterologous protein is expressed as a fusion protein with the ligand-binding domain of the estrogen receptor. The fusion protein, that is constitutively expressed, remains in an inactive state, due to the binding of chaperons of the Hsp90 family. The repression is reversed by adding hormone. (B) Modular structure of Raf and ΔRaf:ER, a conditionally active form of Raf-1. (C) Biologically active ΔRaf-1:ER triggers phosphorylation and activation of ERK2. HT22 cells, murine cells of hippocampal origin, and HT22-ΔRaf-1:ER cells were treated with 4OHT (+) or left untreated (-). Whole cell extracts were prepared 15 min after stimulation and subjected to Western blot analysis. The blots were incubated with a rabbit antibody directed against the phosphorylated form of ERK1/2.

Alternatively, expression of the catalytic domain of either Raf-1, B-Raf, or A-Raf as a fusion protein with the hormone binding domain of the estrogen receptor [ER] generates a hormone-regulated constitutively active Raf. The ΔRaf:ER fusion protein remains in an inactive state in the absence of hormone, but is rapidly activated by the addition of hormone [13]. Figures 2A and 2B outline the strategy of using steroid-binding domains for regulating the function of Raf *in cis *[14]. In the absence of hormone, a heat-shock protein such as Hsp90 binds to the estrogen receptor domain and inhibit the catalytic function of the Raf-estrogen receptor fusion protein by steric hindrance, thus keeping the protein in an inactive state. Treatment with estrogen triggers the dissociation of the heat-shock proteins, leading to a reversal of repression. As a result, Raf activity can be hormonally controlled. The use of the estrogen receptor mutant ER^Tamoxifen Mutant ^allows the use of the synthetic ligand, 4-hydroxytamoxifen (4OHT) for induction. The encoded hormone binding domain of the estrogen receptor contains a glycine residue at position 525, instead of an arginine. As a result, the receptor is largely insensitive to 17β-estradiol, but is readily activated by 4OHT [15]. The activation of the ERK signaling pathway can be visualized by Western blot analysis using antibodies that specifically recognize the phosphorylated forms of ERK1 and ERK2 (figure 2C). In this review, we will summarize some of the findings obtained with cells expressing Raf-estrogen receptor fusion proteins, which we consider the most important ones.

### Role of Raf in the regulation of proliferation

The fact that Raf is activated following stimulation of the cells with mitogens (i.e. EGF, PDGF, IGF) indicates that these enzymes are involved in the regulation of cell growth and proliferation. Accordingly, expression of the hormone-regulated form of Raf-1, ΔRaf-1:ER, induced cell proliferation in NIH 3T3 fibroblasts that was accompanied by an upregulation of cyclin D1 and a repression of p27^KIP^, a cyclin-dependent protein kinase inhibitor [16]. Expression of conditionally active forms of A-Raf and B-Raf in NIH 3T3 cells revealed differences between the individual Raf isoforms. While the activation of both ΔA-Raf:ER and ΔB-Raf:ER induced the activation of MEK and ERK protein kinases, ΔB-Raf:ER activated MEK with the highest efficiency [17]. A microarray analysis performed with human epithelial cells underlined the importance of MEK activation by Raf [2]. Moreover, the fact that activation of ΔRaf:ER strongly induced the expression of growth factors of the EGF growth factor family suggests the existence of an autocrine loop through the activation of the EGF receptor: Activation of ΔRaf:ER triggers the stimulatation of the EGF receptor. As a result, the Raf-MEK-ERK signaling pathway is activated, leading to further synthesis of EGF growth factors [2,18].

**Figure 3:**
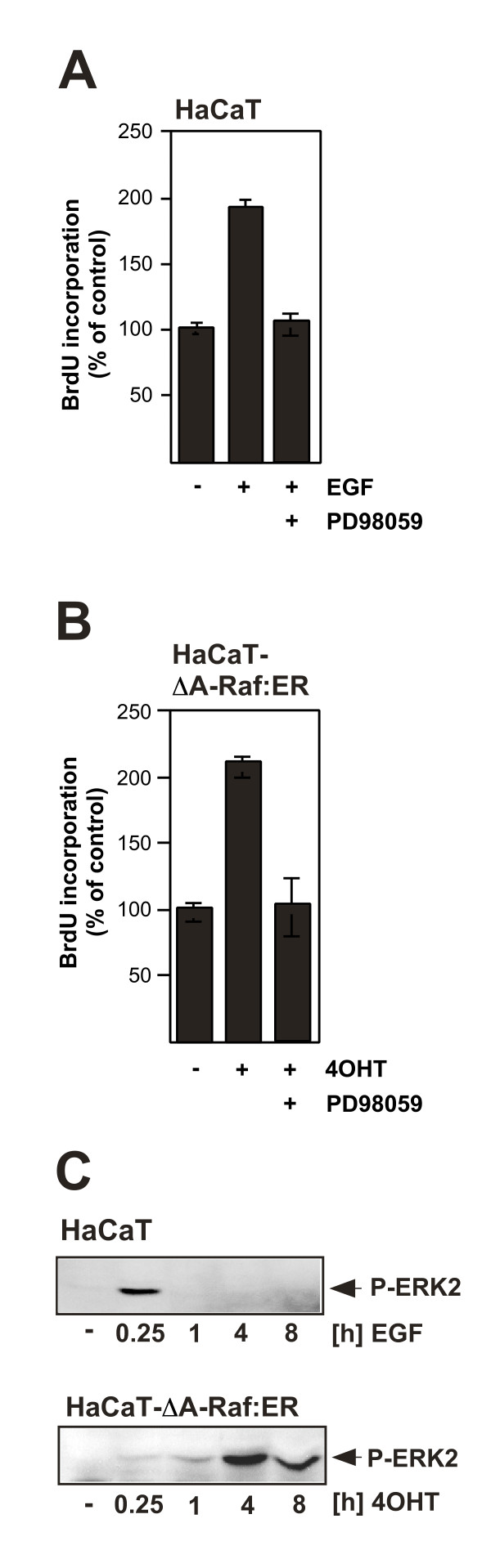
**Activation of a conditionally active form of A-Raf(ΔA- Raf:ER) induces proliferation of human HaCaT keratinocytes via activation of the ERK signaling pathway**. (A, B) Stimulation of HaCaT-ΔA- Raf:ER cells with either EGF (1 ng/ml) (A) or 4OHT (25 nM) (B) leads to an upregulation of DNA synthesis, as measured by the incorporation of the pyrimidine analogue 5-bromo-2'-deoxyuridine (BrdU) instead of thymidine into the DNA of proliferating cells. The incorporated BrdU was detected by immunoassay. Mitogenic signaling induced by EGF or 4OHT was completely abrogated by the MAP kinase kinase inhibitor PD98059. (C) Kinetics of ERK activation in EGF and 4OHT treated HaCaT-ΔA-Raf:ER cells. Whole cell extracts were prepared from cells at different time points and subjected to Western blot analysis. The blots were incubated with an affinity purified rabbit antibody directed against the phosphorylated (active) form of ERK2 (reproduced from [19] with copyright permission from the American Physiological Society).

In keratinocytes, activation of the EGF receptor triggers proliferation of the cells and involves the ERK signaling pathway (figure 3A) [19]. The importance of the ERK signaling pathway for growth of human keratinocytes was further demonstrated with HaCaT keratinocytes expressing ΔA-Raf:ER. Proliferation of the cells was induced with 4OHT and completely inhibited by pretreatment with the MAP kinase kinase inhibitor PD98059 (figure 3B), indicating that the mitogenic activity of ΔA-Raf:ER is mediated by the activation of ERK. A comparison between the kinetics of ERK phosphorylation and activation by EGF or 4OHT revealed major differences in the duration of ERK activation (figure 3C). While EGF induced a strong phosphorylation of ERK within 1 hour after stimulation, phosphorylated ERK was barely detected in ΔA-Raf:ER expressing keratinocytes that had been incubated for 1 hour with 4OHT. Phosphorylated ERK was, however, detected in these cells 4 and 8 hours after induction of ΔA-Raf:ER. These results indicate that the kinetics of ERK activation (transient versus sustained) are of minor importance for the induction of the mitogenic program of keratinocytes by A-Raf. This observation is in contrast to the role of ERK in neuronal survival where a sustained activation of ERK is required for neuroprotection (see below).

In a human breast epithelial cell line, activation of ΔRaf:ER triggered the expression of genes encoding regulators of cell proliferation, including cyclin D1, and induced a transient increase in S phase cells. However, Raf activation did not induce growth factor-independent proliferation [18], in contrast to the situation encountered with ΔA-Raf:ER expressing keratinocytes. These data indicate that cell-type specific variations are important for the biological outcome of Raf activation.

Although the activation of a conditional form of Raf can promote DNA synthesis and cellular proliferation, other reports show that it can also provoke cell cycle arrest. Expression of ΔRaf-1:ER in small lung cancer cells induced a growth inhibitory pathway that is accompanied by the induction of the cyclin-dependent protein kinase inhibitor p27^KIP ^and a decrease in cdc2 protein kinase activity [20]. In prostate cancer cells, activation of ΔRaf-1:ER induced expression of the cyclin-dependent protein kinase inhibitor p21^KIP ^and an accumulation of the cells in G1, thus leading to growth suppression [21]. Likewise, ΔRaf-1:ER and ΔB-Raf:ER elicited a G1 arrest in NIH 3T3 cells that was accompanied by an upregulation of the cyclin-dependent protein kinase inhibitor p21^KIP^. In contrast, activation of ΔA-Raf:ER promoted the entry of quiescent NIH 3T3 cells into the S-phase of the cell cycle. A catalytically potentiated form of ΔA-Raf:ER, however, induced cell cycle arrest and enhanced p21^KIP ^expression, similarly to ΔB-Raf:ER or ΔRaf-1:ER [22]. These data suggest that the catalytical activity and the duration of the signaling of Raf might determine the role of these enzymes in the progression of the cell cycle. In addition, cell type-specific differences are essential for Raf induction and impairment of the growth capacity of the cells.

### Anti-apoptotic role of Raf

Raf-1-deficient embryos are growth retarded and apoptotic cells are found in different tissues [6,7]. Raf-1-deficient fibroblasts are hypersensitive to apoptotic stimuli such as serum withdrawal or Fas/Fas ligand interaction. Thus, it was concluded that the major function of Raf-1 is to counteract apoptosis [7]. Also B-Raf-deficient embryos die because of vascular defects due to apoptotic death of differentiated endothelial cells [23].

The activation of the MEK/ERK signaling pathway by Raf has been correlated with inhibition of programmed cell death. The ERK signaling pathway has been described to play an important role as a main antagonist of various apoptosis-inducing challenges [[24,25]; reviewed in ref [26]]. Activation of the ERK signaling pathway suppresses the proapoptotic activity of stress-activated JNK/p38 protein kinases in PC12 pheochromocytoma cells, thus protecting the cells from NGF withdrawal-induced cell death [27]. In line with this, BDNF-elicited ERK activation protects cortical neurons against a challenge with the topoisomerase I inhibitor campthothecin [28]. In addition, it has been shown that activation of the ERK signaling pathway via treatment of the cells with either EGF or 12-*O*-tetradecanoylphorbol-13-acetate may lead to an inactivation of caspase-9 due to a direct phosphorylation of Thr125 of caspase-9 by ERK. This phosphorylation blocks caspase-9 processing and the subsequent activation of caspase-3 [29].

The survival of cells requires the presence of survival factors, and the lack of this trophic support is one of the best-studied signals for induction of cell death. In Rat-1 fibroblasts, overexpression of B-Raf protected the cells from apoptosis, induced by growth factor withdrawal. Treatment with the MEK inhibitor PD98059 blocked the anti-apoptotic activity of B-Raf, indicating that the activation of the Raf-MEK-ERK signaling pathway is necessary for the anti-apoptotic role of B-Raf in Rat-1 fibroblasts [30].

**Figure 4:**
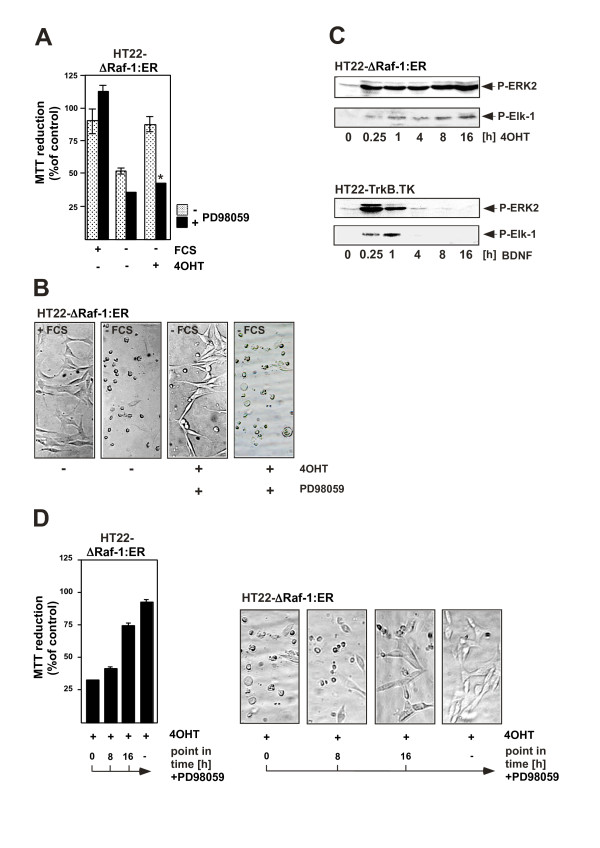
**A conditionally active form of Raf-1 (ΔRaf-1:ER) protects HT22 neuronal cells against serum deprivation-induced cell death via activation of ERK**. (A, B) Activation of Raf-1 provides protection against serum deprivation-induced apoptosis. HT22-ΔRaf-1:ER cells were serum-starved in the presence or absence of 4OHT (200 nM) for twenty-four hours. The reduction of colorless 3-(4,5-dimethylthiazol-2-yl)-2,5-diphenyltetrazolium bromide by mitochondrial NAD(P)H-dependent dehydrogenases to formazan dye crystals was used as an indicator for cell survival (MTT assay). (A); ⋆, values statistically significantly different (p < 0.005) from those of cells incubated in the absence of serum and PD98059, but in the presence of 4OHT. (B) HT22ΔRaf-1:ER cells or HT22pac cells cells were serum-starved in the presence or absence of 4OHT for twenty-four hours. The cytotoxic effect of serum withdrawal was determined by phase contrast microscopy. The neuroprotection was abrogated by incubation with the MEK inhibitor PD98059, indicating that the ERK signaling pathway is essential for ΔRaf-1:ER mediated neuroprotection. (C) Kinetics of ERK2 and Elk-1 phosphorylation after stimulation with BDNF or 4OHT. BDNF stimulation triggers a rapid, but transient activation of ERK in TrkB receptor expressing HT22 cells (HT22-TrkB.TK cells, upper panel), whereas activation of the ΔRaf-1:ER fusion protein leads to a sustained activation of ERK, lasting for hours (HT22-ΔRaf-1:ER cells, lower panel). The kinetics of ERK phosphorylation and activation correlate very well with phosphorylation and activity of the ternary complex factor Elk-1, a nuclear substrate of ERK that functions as a key regulator of serum response element-driven gene transcription. (D) Prolonged activation of ERK is essential for neuroprotection. HT22 cells expressing ΔRaf-1:ER were serum-starved and incubated with 4OHT for twenty-four hours. PD98059 was added simultaneously (t = 0), or eight (t = 8) or sixteen hours (t = 16) after stimulation of the cells with 4OHT. Cells were analyzed twenty-four hours after stimulation by either the MTT assay (left) or by phase contrast microscopy (right) (reproduced from [31] with copyright permission from Blackwell Publishing, Oxford).

Experiments using HT22 immortalized neurons derived from the hippocampal region of the CNS showed that stimulation with BDNF rescues the cells from serum withdrawal-induced cell death when the BDNF receptor TrkB is expressed. An analysis of intracellular signaling cascades revealed that stimulation of the TrkB receptor with BDNF leads to an activation of both the ERK and the PI3 kinase pathways. A pharmacological approach showed that the major neuronal survival-promoting signaling pathway includes an activation of PI3 kinase and AKT [31]. These and other observations [28] indicate that the signaling cascade BDNF → TrkB stimulation → PI3 kinase activation → activation of AKT →→ cell survival is of general importance and is not limited to a particular neuronal population or neuronal cell line. In contrast, the neuroprotective activity of BDNF is independent of the ERK signaling pathway since PD98059 did not impair the BDNF-mediated protection of neurons against serum withdrawal-induced programmed cell death. To clarify the role of ERK for neuroprotection, HT22 cells were analyzed that expressed a ΔRaf-1:ER fusion protein and allowed the selective activation of the ERK signaling pathway (figure 2C). Activation of the catalytic function of Raf-1 by 4OHT rescued HT22 cells from serum withdrawal-induced cell death, as depicted in figure 4A. The neuroprotective role of ΔRaf-1:ER was confirmed by phase contrast microscopy (figure 4B). Inhibition of ΔRaf-1-induced MEK activation by PD98059 blocked the cytoprotective activity of ΔRaf-1:ER [31], indicating that the activation of ERK via MEK is the underlying cause for neuroprotection mediated by the activation of the ΔRaf-1-ER fusion protein. These results were a puzzle to be solved: BDNF-mediated neuroprotection against serum withdrawal-mediated cell death was independent of ERK; in contrast, the Raf-1-estrogen receptor fusion protein protected the cells solely via activation of the ERK signaling pathway. To find clues to the solution of the puzzle, the kinetics of ERK activation in HT22 cells have been investigated (figure 4C). Stimulation of TrkB expressing HT22 cells with BDNF triggered a robust, but transient activation of ERK that was not sufficient to confer protection against the loss of trophic support. In contrast, expression of a conditionally activatable Raf-1 that induced a sustained ERK phosphorylation lasting for hours, rescued neuronal HT22 cells from serum deprivation-induced cell death. The phosphorylation state of the ERK substrate Elk-1 mirrored the kinetic profile of ERK activation, i.e. transient or sustained activation of ERK is translated in the nucleus into a transient or sustained activation of Elk-1 (figure 4C). We then asked the question how long ERK has to be activated in order to protect HT22 cells from serum deprivation-induced apoptosis. Addition of PD98059 at different time points following treatment of the cells with 4OHT revealed that a prolonged activation of ERK, lasting for hours, is necessary for neuroprotection [31] (figure 4D). These results shed light on the fact that the kinetics of ERK activation (transient versus sustained) are of major importance for the neuroprotective activity. Thus, activation of PI3 kinase by BDNF in neuronal cells represents the dominant survival pathway, whereas the ERK signaling pathway plays no or only a marginal role. However, a sustained activation of ERK, lasting for several hours, protects neurons from growth factor deprivation-induced cell death, indicating that the duration of ERK activation is of major importance for its neuroprotective biological function. Thus, stimulation of the cells with neurotrophins of elevated concentrations, or stimulation with several ligands, i.e. BDNF and EGF, may trigger a sustained activation of ERK.

The activation of an estrogen-inducible activated Raf-1 mutant ΔRaf-1:ER also prevented apoptosis induced by loss of matrix contact (anoikis), cytoskeletal integrity and serum removal in lung fibroblasts [32]. In these cells it has been shown that activation of ΔRaf-1:ER prevented the upregulation of Bim, a proapoptotic BH3-only protein of the Bcl-2 family, in serum-starved cells. This rescue relies on the activation of the ERK pathway and was independent of the JNK → c-Jun and PI3 kinase → PDK → AKT pathway [33]. In human breast epithelial cells, the expression of genes encoding growth factors of the EGF family as a result of ΔRaf-1:ER activation protected the cells from detachment-induced apoptosis [18]. Activation of ΔRaf-1:ER also blocked programmed cell death induced by TGFβ in MLCK epithelial cells [34]. However, activation of ΔRaf-1:ER did not provide protection against oxidative glutamate toxicity in HT22 hippocampal cells [35]. These data indicate that the anti-apoptotic function of Raf is restricted to particular apoptotic signaling pathways.

In addition to the well-established target MEK, Raf may use other effectors to inhibit programmed cell death. It has been shown that Raf-1 promotes cell survival in a MEK/ERK-independent manner via antagonizing apoptosis signal-regulating kinase-1 (ASK-1) [36]. Raf-1 is also targeted to the mitochondria by Bcl-2 that leads to cell survival without ERK activation, probably by phosphorylating substrates other than MEK, such as Bcl-2 family members [37,38].

### Role of Raf in cellular differentiation

**Figure 5:**
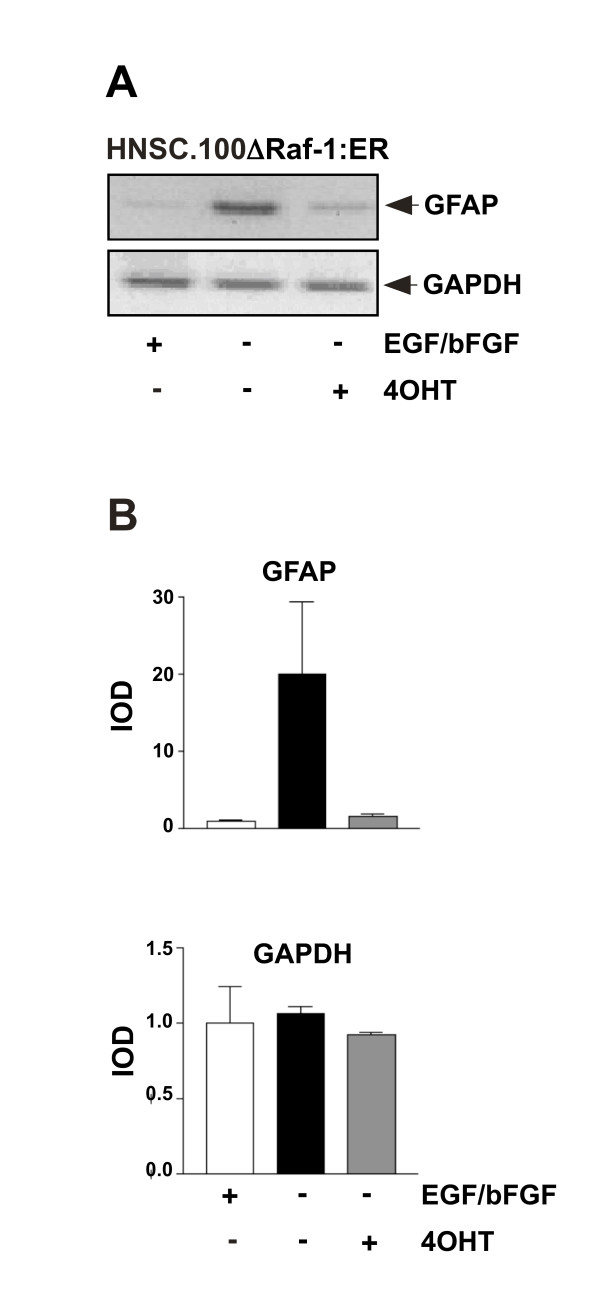
**Activation of ΔRaf-1:ER blocks differentiation along the astrocytic lineage in human neural stem cells**. (A) HNSC.100 neural stem cells expressing ΔRaf-1:ER were incubated with or without EGF, bFGF and 4OHT, as indicated. RNA was isolated and analyzed by RT-PCR. (B) Quantification and statistical analysis of the data shown in (A) (GFAP, glial fibrillary acidic protein; GAPDH, glycerinaldehyde-3-phosphate-Dehydrogenase; IOD = Integrated optical density) (reproduced from [43], copyright by the American Society for Biochemistry and Molecular Biology).

Raf activation has been discussed primarily as an integral part of the ERK signaling pathway controlling cellular growth. The impact of Raf in the control of cellular differentiation has only been put in the limelight in recent years. The lethal phenotypes observed in either Raf-1-, A-Raf- or B-Raf-deficient mice sheds light on the essential role of Raf during development. Nullizygous B-Raf^-/-^-embryos, for example, die by embryonic day 12.5 showing defects in vascular endothelial cell differentiation [23]. In cell culture models, B-Raf controls myelopoiesis at multiple stages. In particular, B-Raf deficient ES cells have a quantitative defect in myeloid progenitor cell formation [39]. B-Raf is also crucial for T-cell development, in particular for the transition to CD4^+ ^and CD8^+ ^single-positive cells [40]. In immortalized cells from rat hippocampus neurons, activation of ΔRaf-1:ER was shown to induce neuronal differentiation [41]. In neural stem cells, supplementation of the medium with EGF and bFGF is necessary to inhibit differentiation. Removal of the mitogens stops the cell cycle and induces differentiation [42]. Figure 5 shows that expression of the astrocytic marker GFAP is upregulated in differentiated HNSC.100 neural stem cells. This upregulation of GFAP expression was prevented in ΔRaf-1:ER-expressing neural stem cells that were stimulated by 4OHT in the absence of EGF and bFGF in the medium [43], indicating that enhanced Raf-1 activity blocked the differentiation of the cells. Together, these data show that Raf influences developmental processes, although the exact molecular mechanisms have to be determined in each cell type.

## Conclusion

Results obtained with constitutively active Raf mutants has been questioned, since the lack of the regulatory domains in the Raf mutants may compromise the substrate specificity and the dynamic regulation of activity [9]. Nevertheless, the many data obtained using these mutants have improved our knowledge of the functions of Raf in growth control, apoptosis and differentiation.

## Abbreviations

ER: estrogen receptor; ERK: extracellular signal-regulated protein kinase; 4OHT: 4-hydroxytamoxifen.

## Competing interests

The authors declare that they have no competing interests.

## Authors' contributions

GT drafted and wrote the manuscript. ME and OR have critically revised the manuscript and prepared the figures. All authors read and approved the final manuscript.
